# Renal anemia: from erythropoietin deficiency to erythropoietin resistance

**DOI:** 10.3389/fphar.2026.1928390

**Published:** 2026-10-08

**Authors:** Aojiao Chu, Jinyang Bu, Xinfu Zou, Zheng Zhang, Qin Zheng, Ling Shi, Meihong Luo

**Affiliations:** Department of Hematology, Baoshan Hospital Affiliated to Shanghai University of Traditional Chinese Medicine, Shanghai, China

**Keywords:** chronic kidney disease, EPO resistance, erythropoietin, ESAs, HIF-PHIs, pharmacology, renal anemia

## Abstract

Renal anemia is one of the core complications of chronic kidney disease (CKD) and is closely associated with poor patient prognosis. Renal interstitial fibrosis or impaired oxygen sensing leads to insufficient erythropoietin (EPO) production, which constitutes the fundamental mechanism initiating renal anemia. On this basis, multiple pathological factors—including the inflammatory microenvironment in CKD, iron deficiency, and mineral and bone disorders—synergistically induce EPO resistance, further exacerbating anemia and diminishing the response to existing therapies. Currently, the treatment of renal anemia has moved beyond simple EPO replacement and is progressively shifting toward a dual strategy that emphasizes both correction of EPO resistance and enhancement of drug responsiveness. This article summarizes the molecular network through which insufficient EPO synthesis and EPO resistance synergistically aggravate renal anemia, systematically reviews the pharmacological basis, limitations, and safety risks of current drug therapies, and discusses future research directions and potential therapeutic targets in this field.

## 1 Introduction

Renal anemia is a common complication of chronic kidney disease (CKD), characterized by reduced red blood cell counts and decreased hemoglobin levels (Improving Global Outcomes Anemia Work Group, 2026). Clinically, hemoglobin <130 g/L in males and <120 g/L in females are generally used as diagnostic thresholds for renal anemia (Improving Global Outcomes Anemia Work Group, 2026). The prevalence and severity of renal anemia increase with CKD progression. Patients in CKD stages 1–2 have relatively mild anemia, whereas more than half of patients in stages 4–5 have anemia, and 90% of end-stage renal disease patients undergoing dialysis require anemia-correcting medications (Fishbane and Coyne, 2020; Hanna et al., 2021). Global epidemiological data show that in 2021, the burden of renal anemia increased by 96.24% compared with 1990, indicating a continuously rising disease burden (Liu et al., 2025b). As an independent risk factor for CKD progression and poor prognosis, effective intervention for renal anemia is critical to improving long-term patient outcomes (Hanna et al., 2021; Romagnani et al., 2025).

The core pathogenic mechanism of renal anemia is insufficient synthesis of erythropoietin (EPO), while EPO resistance is a key reason why anemia is difficult to correct and why treatment responses are poor (Babitt et al., 2026). EPO is the central factor regulating erythrocyte proliferation and differentiation, and is mainly synthesized and secreted by renal interstitial fibroblasts (Ku et al., 2023; Macdougall, 2024). During CKD progression, renal interstitial fibrosis or impaired local oxygen sensing in the kidney leads to reduced EPO production, initiating the development of anemia (Shih et al., 2018; Hanna et al., 2021). Erythropoiesis-stimulating agents (ESAs), which supplement exogenous EPO to correct EPO deficiency, remain the classic first-line therapy for renal anemia (Fishbane and Coyne, 2020; Liu et al., 2025b; Ku et al., 2023; Improving Global Outcomes Anemia Work Group, 2026). However, some CKD patients show low responsiveness to ESA therapy, manifested as insufficient hemoglobin increase or the need for higher doses to reach target levels—a phenomenon termed EPO resistance, which is associated with poor CKD outcomes (Johnson et al., 2007). Inflammatory cytokines, iron metabolism disorders, mineral and bone disorders (MBD), toxin accumulation, malnutrition, gut microbiota dysbiosis, and interference from certain CKD medications are all important factors mediating EPO resistance (Tarng et al., 1999; Anderson et al., 2013; Babitt et al., 2026).

In renal anemia patients with coexisting EPO resistance, simply increasing the ESA dose has limited efficacy; identifying and correcting the triggers of EPO resistance is key to optimizing anemia management (Improving Global Outcomes Anemia Work Group, 2026). Combination therapy with iron agents is a commonly used clinical strategy, but it carries risks of inducing iron overload and exacerbating inflammation and iron metabolic disturbances (Bazeley and Wish, 2022). Hypoxia-inducible factor prolyl hydroxylase inhibitors (HIF-PHIs), which stabilize hypoxia-inducible factor (HIF)-2α, not only stimulate endogenous EPO production but also effectively improve iron metabolism and alleviate inflammation, making them a current research hotspot (Anderson et al., 2013; Locatelli et al., 2022). Adjunctive agents such as anti-inflammatory drugs, SGLT-2 inhibitors, and traditional Chinese medicine are also being gradually applied in clinical practice to overcome EPO resistance.

From a pharmacological perspective, renal anemia is not merely a deficiency disease requiring EPO replacement therapy, but also a complex condition characterized by abnormal drug responses. Current domestic and international reviews mostly address EPO deficiency or EPO resistance separately, lacking a systematic synthesis of intersecting pathways, which fails to meet the needs of pharmacological research and clinical medication guidance. This review focuses on the pathological evolution from EPO deficiency to EPO resistance, summarizing the pharmacological targets and therapeutic limitations of existing drugs. In addition, we discuss how emerging therapeutic targets might overcome the shortcomings of conventional EPO replacement therapy, aiming to provide a theoretical basis for precision pharmacological intervention and individualized management of renal anemia.

We searched PubMed and Web of Science from database inception to August 2026, and simultaneously queried ClinicalTrials.gov, the U.S. National Institutes of Health clinical trial registry. Search terms included “chronic kidney disease,” “renal anemia,” “EPO,” “EPO resistance,” “ESAs,” “HIF-PHI,” “iron metabolism,” “hepcidin,” “inflammation,” “CKD-MBD,” “Fibroblast growth factor 23 (FGF23),” “uremic toxins,” “long non-coding RNAs (lncRNAs),” “hepcidin-targeting agents,” “IL-6 monoclonal antibodies,” and related terms. Additionally, we manually screened the reference lists of all included review articles to identify further potentially eligible studies. We synthesised mechanistic evidence from preclinical studies (both *in vitro* and *in vivo*) to clarify the pathophysiology of EPO deficiency and EPO resistance in CKD. For clinical evidence, we referenced clinical guidelines such as KDIGO and included RCTs, meta-analyses, and real-world observational studies, with particular emphasis on the pharmacological mechanisms and clinical data for renal anemia treatments.

## 2 Physiological mechanisms of EPO in regulating erythropoiesis

Erythropoiesis is a precisely orchestrated process in which hematopoietic stem cells (HSCs) differentiate stepwise into red blood cells (RBCs). Erythropoietin (EPO) and its receptor (EPOR) are involved throughout erythroid development and also regulate the homeostasis of the hematopoietic microenvironment. The integrity of this pathway is fundamental to normal hematopoiesis.

### 2.1 The complete process of erythroid cell differentiation

Under the regulation of transcription factors, HSCs differentiate into common myeloid progenitors (CMPs), which then give rise to megakaryocyte-erythrocyte progenitors (MEPs). In the presence of stem cell factor (SCF), MEPs sequentially differentiate into burst-forming unit-erythroid (BFU-E) and colony-forming unit-erythroid (CFU-E). CFU-E can further mature into morphologically identifiable erythroid precursor cells. CFU-E and erythroid precursor cells surround central macrophages to form erythroblastic islands (EBIs). Within this specialized niche of EBIs, erythroid nuclei gradually condense and hemoglobin progressively accumulates, as cells differentiate from proerythroblasts (Pro-E) through basophilic erythroblasts (Baso-E), polychromatic erythroblasts (Poly-E), and orthochromatic erythroblasts (Ortho-E). Ultimately, Ortho-E extrude their nuclei to become reticulocytes (RET), which then detach from EBIs and mature into RBCs(Tichil et al., 2024; Romano et al., 2022; Koury and Haase, 2015; Chasis and Mohandas, 2008).

EPO is the core endocrine factor governing erythropoiesis (Ku et al., 2023). Macrophages serve as key supporting cells in constructing the hematopoietic microenvironment (Li et al., 2020b). As illustrated in Figure 1, EPO binds specifically to EPOR on erythroid cells and macrophages, coordinating erythropoiesis through multiple signaling pathways and across multiple cell types (Li et al., 2020b; Tichil et al., 2024).

**FIGURE 1:**
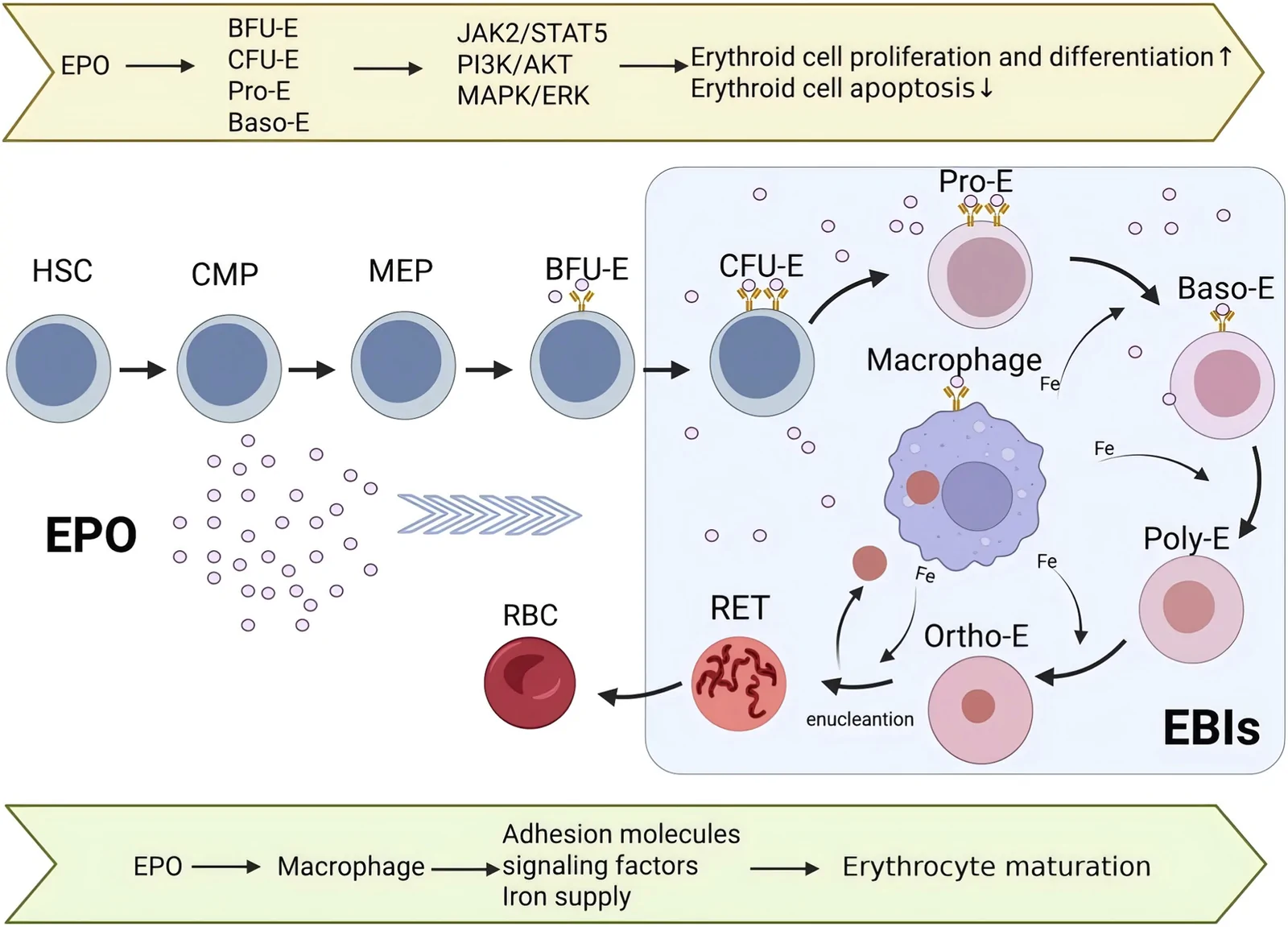
EPO regulates erythroid precursor cells and macrophages to promote erythropoiesis. EPO exerts its regulatory effects on erythropoiesis by binding to EPOR on BFU-E, CFU-E, Pro-E, Baso-E, and macrophages. In erythroid cells, EPO activates the JAK2/STAT5, PI3K/AKT, and MAPK/ERK pathways, promoting proliferation and differentiation while inhibiting apoptosis. In macrophages, EPO upregulates the expression of adhesion molecules and signaling factors, enhances iron supply, and facilitates erythrocyte maturation. EPO, Erythropoietin; HSC, hematopoietic stem cell; CMP, common myeloid progenitor; MEP, megakaryocyte-erythrocyte progenitor; BFU-E, burst-forming unit-erythroid; CFU-E, colony-forming unit-erythroid; Pro-E, proerythroblast; Baso-E, basophilic erythroblast; Poly-E, polychromatic erythroblast; Ortho-E, orthochromatic erythroblast; RET, reticulocyte; RBC, red blood cell; JAK2, Janus kinase 2; STAT5, Signal transducer and activator of transcription 5; PI3K, phosphatidylinositol 3-kinase; MAPK, mitogen-activated protein kinase.

### 2.2 EPO directly regulates proliferation and differentiation of erythroid precursor cells

EPOR is mainly expressed on erythroid precursor cells, with peak expression at the CFU-E and Pro-E stages, making these cells highly sensitive to EPO(Broudy et al., 1991; Nandakumar et al., 2016). EPO binding to EPOR on the cell surface activates Janus kinase 2 (JAK2) tyrosine kinase, which then phosphorylates tyrosine residues in the cytoplasmic domain of EPOR. This recruits and activates signal transducer and activator of transcription 5(STAT5), phosphatidylinositol 3-kinase (PI3K), and mitogen-activated protein kinase (MAPK). Among these, the JAK2/STAT5 pathway predominates, driving proliferation, differentiation, and survival of erythroid precursor cells by upregulating c-myc, the anti-apoptotic gene Bcl-xL, and Spi2A (Prade-Houdellier et al., 2007; Bondurant et al., 1996; Ma et al., 2014; Dev et al., 2013; Lim et al., 2011). PI3K, through activation of AKT kinase, induces the synthesis of cyclins D1 and D3 and the degradation of the cyclin-dependent kinase inhibitor p27, thereby promoting the clonal proliferation of erythroid precursor cells (Bouscary et al., 2003). The MAPK/ERK pathway, via the RAS-MEK-ERK cascade, upregulates erythroid transcription factors GATA-1 and NF-E2, initiating the terminal erythroid differentiation program that generates mature RBCs(Schmidt et al., 2004).

In addition to regulating cell proliferation, EPO also enhances iron utilization by erythroid cells. Through the above-mentioned signaling, erythroid precursor cells increase secretion of erythroferrone (ERFE) and growth differentiation factor 15 (GDF15), which suppress hepatic hepcidin production by blocking the binding of bone morphogenetic proteins (BMPs) to BMP receptors. This in turn increases ferroportin (FPN) expression, enhancing iron uptake by erythroid precursors to meet the hemoglobin synthesis demands of late-stage erythroblasts (Zhang and Enns, 2018; Koury and Haase, 2015) Dysregulation of this pathway represents an important physiological basis for functional iron deficiency and diminished EPO efficacy in CKD, and also constitutes a therapeutic target for subsequent iron supplementation and hepcidin-targeting agents.

### 2.3 EPO acts on macrophages to remodel the hematopoietic microenvironment

Macrophages associate with erythroblasts to form EBIs, which constitute the core of the hematopoietic microenvironment (Borges and Sesti-Costa, 2022; Li et al., 2020b; Wei et al., 2019). EPO binds to EPOR on EBI macrophages, upregulating the expression of the adhesion molecule Vcam1, thereby enhancing macrophage–erythroblast adhesion and maintaining EBI structural integrity. Simultaneously, EPO upregulates macrophage expression of the phagocytosis-related molecule Tim4 and the heme-metabolic molecules Spic and Hmox1, promoting macrophage-mediated phagocytosis and degradation of senescent RBCs and increasing iron recycling (Li et al., 2019). Under stress conditions, the EPO/STAT5 signaling pathway induces macrophages in splenic EBIs to secrete prostaglandin J2 (PGJ2) and prostaglandin E2 (PGE2), promoting differentiation of stress erythroid precursors in the spleen (Chen et al., 2020).

In healthy individuals, EPO maintains hematopoietic homeostasis through direct regulation of erythroid cells and indirect modulation of the hematopoietic microenvironment. However, in the CKD setting, insufficient EPO synthesis and EPO resistance mediated by blockade of the EPO/EPOR pathway synergistically disrupt the erythropoietic network. Therefore, both EPO replacement therapy and restoration of EPO sensitivity represent core directions for pharmacological.

## 3 Molecular mechanisms of CKD-Mediated insufficient EPO synthesis

In adults, 90% of EPO is secreted by a specific subset of interstitial fibroblasts located in the renal cortex and the corticomedullary junction, which are defined as renal erythropoietin-producing cells (REPCs) (Creangă et al., 2025; Liu et al., 2018). Under physiological conditions, hypoxia or anemia induces compensatory EPO synthesis by REPCs. In CKD, however, REPCs transdifferentiate into myofibroblasts and lose their EPO-producing capacity (Sato et al., 2019). Concurrently, disruption of the intrarenal oxygen gradient, coupled with a pathological microenvironment characterized by inflammatory cytokines and accumulation of uremic toxins, collectively impairs the HIF oxygen-sensing pathway and suppresses *Epo* gene transcription. The above pathological mechanisms are illustrated in Figure 2 (Eckardt and Kurtz, 2005; Tomc and Debeljak, 2021).

**FIGURE 2:**
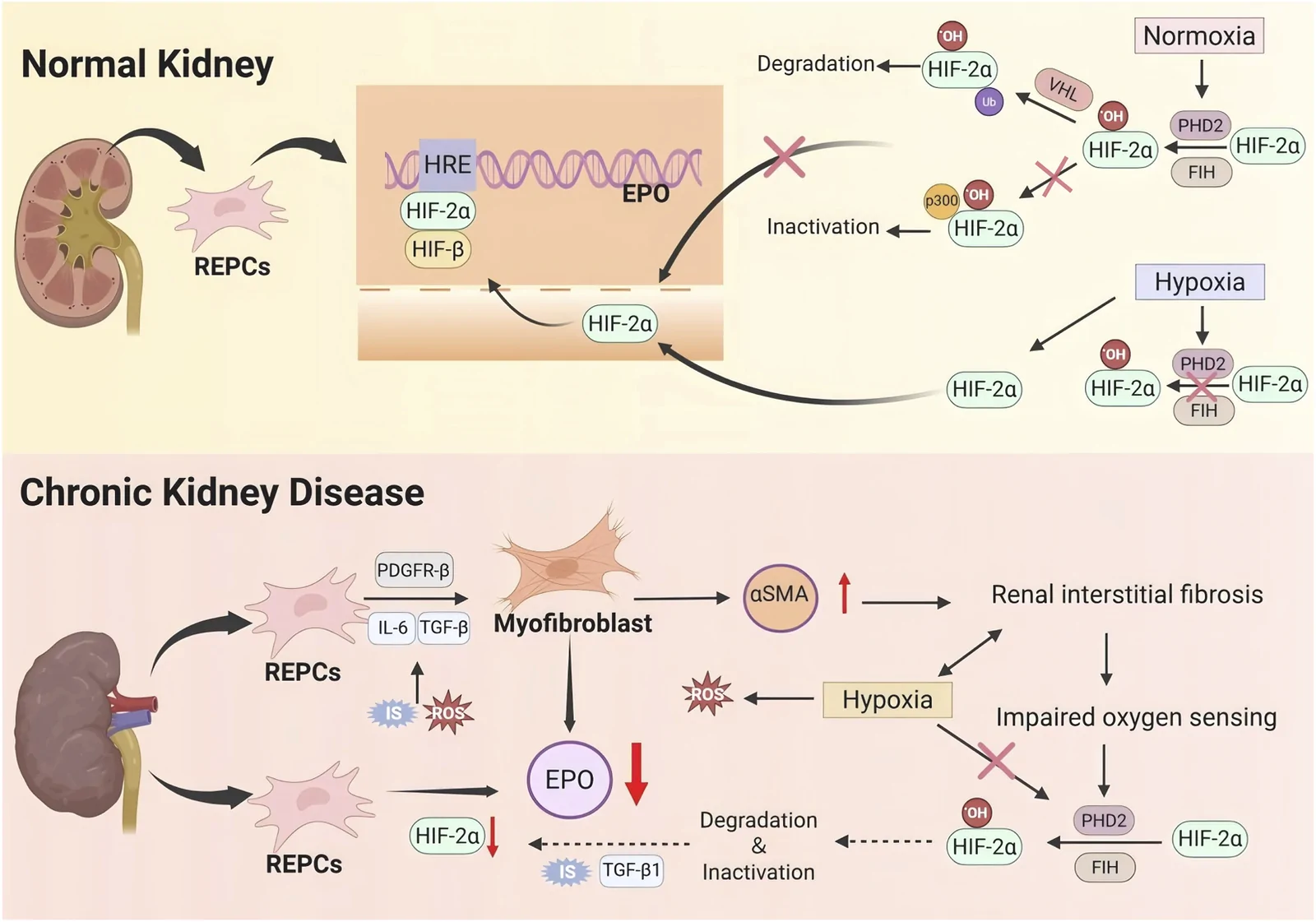
Insufficient EPO synthesis contributes to renal anemia. Under physiological conditions, hypoxia reduces the activities of PHD2 and FIH, leading to the stable expression of HIF-2α, which in turn induces REPCs to compensatorily synthesize EPO. In CKD, however, PDGFR-β, along with inflammatory cytokines (such as IL-6 and TGF-β), IS and ROS, promotes the transdifferentiation of REPCs into myofibroblasts. This results in decreased EPO synthesis and increased αSMA expression, thereby accelerating the progression of renal interstitial fibrosis. The resulting fibrosis disrupts the intrarenal oxygen gradient. Combined with the pathological microenvironment characterized by inflammatory factors and accumulated uremic toxins, this disruption impairs the HIF-2α-mediated oxygen-sensing pathway, ultimately inhibiting EPO gene transcription. EPO, Erythropoietin; REPCs, renal erythropoietin-producing cells; HRE, hypoxia-responsive element; HIF, hypoxia-inducible factor; PHD, prolyl hydroxylase domain; FIH, factor inhibiting HIF; VHL, Von Hippel-Lindau; PDGFR-β, platelet-derived growth factor receptor-β; IL-6, interleukin-6; TGF-β, transforming growth factor-β; IS, indoxyl sulfate; ROS, reactive oxygen species; αSMA, α-smooth muscle actin.

### 3.1 Renal interstitial fibrosis drives the transdifferentiation of REPCs into myofibroblasts

Renal interstitial fibrosis is a hallmark pathological change in CKD (Humphreys, 2018). Lineage tracing studies have confirmed that REPCs, which originate from FoxD1^+^ cells, are an important source of myofibroblasts under CKD conditions (Humphreys, 2018; Chang et al., 2016). Aberrant activation of specific signaling pathways in CKD drives the transdifferentiation of REPCs into myofibroblasts, while progressive fibrosis in turn disrupts the survival microenvironment of REPCs and accelerates their phenotypic abnormalities, ultimately leading to reduced EPO synthesis and aggravated anemia.

In the CKD setting, persistent activation of platelet-derived growth factor receptor-β (PDGFR-β) induces REPC transdifferentiation into myofibroblasts. This phenotypic conversion is often accompanied by hypermethylation of the *Epo* gene promoter region, which silences *Epo* transcription. Although animal studies have demonstrated that the PDGFR-β inhibitor imatinib can reverse these abnormalities and correct anemia (Buhl et al., 2020; Sato et al., 2019), this drug has not yet been applied clinically due to its inhibition of hematopoietic factors and potential nephrotoxicity (Ortiz, 2020).

Various pathological products generated by renal interstitial fibrosis can further promote REPC transdifferentiation into myofibroblasts. Fibrosis disrupts the renal microcirculation, leading to local hypoxia and accumulation of reactive oxygen species (ROS), which triggers oxidative stress and mitochondrial dysfunction (Wang et al., 2022; Aranda-Rivera et al., 2021). These changes, together with the uremic toxin indoxyl sulfate (IS), synergistically promote the release of inflammatory cytokines (Shimizu et al., 2011). Inflammatory cytokines such as interleukin-6 (IL-6) and transforming growth factor-β (TGF-β) activate NF-κB and SMAD signaling pathways, cooperatively driving REPCs toward a myofibroblast phenotype expressing α-smooth muscle actin (αSMA) (Souma et al., 2013).

Therefore, under the combined action of multiple pathological factors in CKD, functional REPCs undergo a reduction in number and phenotypic abnormalities, resulting in a marked decline in EPO-synthesizing capacity (Faquin et al., 1992). Clinical observations also support this mechanism: as renal function declines, renal tissue fibrosis worsens in CKD patients, peripheral blood levels of inflammatory cytokines IL-6, IL-8, and tumor necrosis factor-α(TNF-α) increase simultaneously, serum EPO levels progressively decrease, and anemia gradually worsens (Genovese et al., 2016; Keithi-Reddy et al., 2008). Therefore, protecting REPCs from fibrosis-driven phenotypic conversion may represent a novel therapeutic target for CKD-associated anemia.

### 3.2 Impaired renal oxygen sensing suppresses HIF-2α-mediated EPO transcription

The HIF pathway, which is regulated by oxygen concentration, is the core transcriptional pathway controlling the *Epo* gene in REPCs. HIF-2α, which is predominantly expressed in REPCs, is the dominant factor regulating *Epo* production (Rankin et al., 2007). Following heterodimerization with HIF-β, HIF-2α translocates into the nucleus and binds to the pentanucleotide (5′-RCGTG-3′) hypoxia-responsive element (HRE) in the *Epo* gene promoter region to initiate transcription (Shu et al., 2019; Shih et al., 2018).

Prolyl hydroxylase domain enzymes (PHDs) and factor inhibiting HIF (FIH) jointly regulate HIF-2α stability. Under normoxia, PHDs mediate hydroxylation of HIF-2α, which is then recognized by the von Hippel-Lindau (VHL) protein and targeted for ubiquitin-mediated degradation. FIH hydroxylates HIF-2α, impairing its binding to the transcriptional coactivator p300 and reducing its transcriptional activity, thereby maintaining *Epo* transcription at a basal resting level. Under hypoxic conditions, PHD and FIH activities decline, allowing HIF-2α to stabilize and translocate into the nucleus to initiate robust *Epo* transcription—a core compensatory response to hypoxia (Tomc and Debeljak, 2021; Chen et al., 2016; Lando et al., 2002).

Although extensive renal tissue hypoxia exists in CKD, the HIF-2α pathway exhibits a blunted hypoxia response, and CKD-related structural remodeling of the kidney is a key cause of this phenomenon (Cen et al., 2022). Renal interstitial fibrosis disrupts the balance between oxygen supply from peritubular capillaries and oxygen consumption by renal tubules, impairing the corticomedullary oxygen gradient and countercurrent oxygen diffusion shunting. As a result, although REPCs are situated in an overall hypoxic environment, local oxygen-sensing mechanisms fail to be effectively activated (Geis and Kurtz, 2025). Under such pathological hypoxia, PHDs and FIH remain relatively active (Souma et al., 2016; Faivre et al., 2023), continuously mediating HIF-2α protein degradation and functional inactivation. Concurrently, the unique inflammatory and toxin-laden microenvironment in CKD further potentiates the inhibitory effects on the HIF-2α/EPO pathway (Chiang et al., 2011; Zhu et al., 2020). For example, TGF-β1 suppresses HIF-2α transcription through activation of activin receptor-like kinase-5 (ALK5) (Shih et al., 2021), while the uremic toxin IS downregulates HIF-2α transcriptional activity and inhibits *Epo* gene expression by interfering with HIF-2α–HIF-β heterodimerization (Asai et al., 2016).

HIF-PHIs target and inhibit PHDs, thereby enhancing HIF-2α stability and increasing *Epo* transcription (Locatelli et al., 2022). However, merely inhibiting PHD activity without ameliorating the high FIH activity and the pathological microenvironment perturbed by inflammation and toxins is unlikely to maximize HIF-2α-driven endogenous EPO synthesis (Chang et al., 2016; Shih et al., 2021). Consequently, in patients with CKD stage 5, who have more severe renal interstitial fibrosis, greater inflammatory burden, and higher toxin levels, higher doses of HIF-PHIs are required to achieve target hemoglobin levels, whereas lower doses are effective in CKD stages 3–4 (Li et al., 2025).

FIH remains highly active under pathological hypoxia in CKD, continuously suppressing HIF-2α transcriptional function. Currently, no clinical FIH inhibitors are available, and small-molecule screening targeting FIH represents a potential breakthrough in this field. Furthermore, frontier studies have revealed that HIF-PHIs fail to activate EPO expression in fibroblasts deep within the renal medulla, yet genetic knockout of PHDs enables widespread *Epo* transcription throughout the kidney. This suggests the existence of unknown, cell-type-specific *Epo* transcriptional regulatory pathways in the renal medulla, and exploring upstream pathways of EPO may provide entirely new directions for the development of next-generation targeted therapeutics (Firmke et al., 2025).

## 4 Pathological mechanisms of multifactorial synergistic induction of EPO resistance

EPO resistance is defined as a pathological state in which CKD patients exhibit reduced responsiveness to ESAs(Babitt et al., 2026). Clinically, it is quantified using the EPO Resistance Index (ERI), calculated as follows: ERI = weekly ESA dose (IU/week)/[body weight (kg) * hemoglobin level (g/dL)]](Zhao et al., 2024). A prospective study involving 1,270 hemodialysis patients demonstrated that higher ERI is associated with increased risks of anemia and mortality](Zhao et al., 2024). Therefore, identifying and specifically alleviating EPO resistance holds significant clinical value for improving anemia and long-term outcomes in CKD patients.

In the CKD setting, multiple pathological factors—including chronic inflammation, iron metabolism disorders, CKD-related mineral and bone disorders (CKD-MBD), uremic toxins, and malnutrition, interact with one another, and are further compounded by interference from certain medications. These factors collectively induce EPO resistance through multiple dimensions, such as EPO/EPOR signaling impairment, disruption of hematopoietic substrate supply, and compromised erythrocyte differentiation and survival, thereby forming a complex pathological network.

### 4.1 The inflammatory microenvironment is the initiating factor of EPO resistance

A persistent inflammatory microenvironment is the primary risk factor for EPO resistance in patients with renal anemia (Linde et al., 1997; Feret et al., 2022). It comprehensively mediates EPO resistance and suppresses hematopoiesis by interfering with the EPO/EPOR signaling pathway, iron metabolism, and erythrocyte quality.

Inflammatory cytokines inhibit EPO/EPOR signal transduction, suppress erythroid differentiation, and promote erythroid apoptosis. IL-1 attenuates the EPO/EPOR-JAK2/STAT5 signaling pathway by reducing EPOR expression on the surface of erythroid precursor cells (Bandach et al., 2021; Wang et al., 2018). TNF-αsilences the *Epo* gene by upregulating microRNA-663 (Ozkurt et al., 2021), while simultaneously blocking the EPO/EPOR pathway, leading to an accumulation of erythroid precursors and reduced numbers of Poly-E and RET, thereby impairing erythroid differentiation (Borin-Crivellenti et al., 2023). In addition, the damage-associated molecular pattern HMGB1 is elevated under inflammatory conditions and directly interferes with EPO-EPOR binding via a RAGE-independent pathway, inhibiting the JAK2-STAT5 and mTOR pathways and inducing apoptosis of erythroid precursors, further amplifying the EPO signaling defect (Dulmovits et al., 2022).

Inflammatory cytokines induce iron-restricted hematopoiesis. IL-6 activates the JAK2–STAT3 signaling pathway to promote hepatic hepcidin secretion (Silvestri et al., 2023; Marques et al., 2025), accelerating ubiquitin-mediated degradation of ferroportin (FPN) (Nemeth and Ganz, 2021). Concurrently, IL-6 recruits histone deacetylases HDAC1 and HDAC3 to the antioxidant response element in the promoter region of the FPN-encoding gene *Slc40a1* via NF-κB pathway activation, thereby suppressing FPN transcription (Marques et al., 2025). Dysregulation of hepcidin and FPN blocks macrophage iron release and intestinal iron absorption, reducing serum iron and transferrin saturation, which renders the erythropoietic effect of EPO ineffective due to restricted iron availability (Yan and Xu, 2020).

The inflammatory microenvironment exacerbates erythrocyte damage and reduces erythrocyte quality. The inflammatory cytokine TGF-β1 induces macrophage polarization toward the M2 phenotype and enhances aberrant macrophage phagocytosis of erythrocytes through the EPO/EPOR-JAK2/STAT3 pathway (Wu et al., 2024; Meng et al., 2024). Under inflammatory stimulation, erythrocytes undergo structural and functional damage, including increased membrane fragility (Stuart et al., 2024), reduced deformability (Silva-Herdade et al., 2016), and shortened lifespan (Meng et al., 2024), which directly attenuate the efficacy of EPO(Linde et al., 1997). Furthermore, reduced erythrocyte deformability increases blood viscosity and raises the risk of thrombosis (Silva-Herdade et al., 2016). Thrombotic cardiovascular and cerebrovascular events represent major safety concerns of ESA or HIF-PHI therapy, significantly compromising patient outcomes (Huang et al., 2023). This suggests that merely raising hemoglobin levels with drugs cannot eradicate the inflammation-driven EPO resistance and erythrocyte quality defects, making it difficult to achieve long-term prognostic improvement.

### 4.2 Iron deficiency impedes hematopoietic progression

Iron is highly involved in erythropoiesis. It is not only an essential substrate for hemoglobin synthesis but also acts as a key cofactor regulating erythroid cell proliferation and differentiation, and participates in EPO signal transduction. Sufficient bioavailable iron is a fundamental prerequisite for maintaining the erythropoietic activity of EPO(Rishi and Subramaniam, 2017). Iron homeostasis is commonly disrupted in CKD patients, constituting an independent risk factor for clinical EPO resistance and progression of refractory renal anemia (Improving Global Outcomes Anemia Work Group, 2026; Bazeley and Wish, 2022).

Iron homeostasis abnormalities in CKD patients can be classified into absolute iron deficiency and functional iron deficiency. Absolute iron deficiency results from inadequate iron intake due to gastrointestinal dysfunction and dietary restrictions, combined with continuous iron loss caused by blood sampling, dialysis, and occult bleeding (Bazeley and Wish, 2022). Functional iron deficiency is a key mechanism of EPO resistance in renal anemia patients and forms a tight pathological loop with the aforementioned inflammatory microenvironment pathway. The hepcidin/FPN axis imbalance induced by microinflammation, together with reduced renal clearance of hepcidin (Lee et al., 2017), leads to impaired iron utilization (Silvestri et al., 2023; Marques et al., 2025; Nemeth and Ganz, 2021).

Both absolute and functional iron deficiency significantly reduce bioavailable iron in the body, thereby exacerbating EPO resistance. Iron deficiency directly inhibits heme synthesis and downregulates globin transcription, resulting in reduced hemoglobin formation and directly impeding erythrocyte maturation (Fujiwara and Harigae, 2025). Moreover, iron deficiency exacerbates erythrocyte damage. Iron is a critical cofactor for glutathione peroxidase 4 (GPX4); iron deficiency reduces GPX4 activity, triggering erythrocyte lipid peroxidation and calcium influx, which accelerate erythrocyte apoptosis and senescence (Deng et al., 2025). The influx of calcium further activates the calcium-dependent protease calpain, which degrades erythrocyte membrane skeletal proteins, disrupts membrane stability, and reduces erythrocyte deformability, aggravating erythrocyte functional defects (Deng et al., 2025). Concurrently, impaired iron utilization can specifically block the EPO/EPOR pathway. Under conditions of restricted iron utilization in erythroid cells, the iron-sensing transferrin receptor 2 detects intracellular iron deficiency signals and reduces Scribble protein expression, thereby decreasing EPOR expression on the erythroid cell surface. Loss of EPOR expression suppresses the JAK2/STAT5 pathway, blocking erythroid proliferation and differentiation, while selectively preserving the Akt survival signaling pathway. This ultimately results in a differentiation blockade characterized by an abnormal increase in erythroid precursors but insufficient generation of mature erythrocytes, inducing refractory EPO resistance (Khalil et al., 2018).

Although ESA therapy accelerates erythropoiesis, it also increases iron consumption (Cappellini et al., 2017). Therefore, simply supplementing with EPO preparations cannot resolve this dilemma; combination therapy with iron agents is a key strategy for optimizing anemia treatment in clinical practice. At the same time, distinguishing between absolute iron deficiency and functional iron deficiency is crucial for individualized iron supplementation and for optimizing ESA dosing.

### 4.3 Mineral and bone disorders interfere with bone marrow hematopoiesis

CKD-MBD is initiated as early as the early stages of CKD. It not only disrupts the bone marrow hematopoietic microenvironment and the EPO/EPOR signaling pathway through multiple mechanisms, but also deeply intertwines with inflammation and iron metabolism disorders, collectively exacerbating EPO resistance and the progression of renal anemia (Hu et al., 2022; Carullo et al., 2024).

FGF23 is a core molecule through which CKD-MBD mediates EPO resistance. As an early biomarker of CKD-MBD (Simic and Babitt, 2021), FGF23 synthesis is promoted by the inflammatory and iron-disordered microenvironment (David et al., 2016; Durlacher-Betzer et al., 2018). Elevated FGF23 negatively regulates hematopoiesis by inhibiting HSC homing, interfering with EPO/EPOR signaling, exacerbating iron disturbances, and reducing erythrocyte quality. On the one hand, FGF23 inhibits HSC homing to the bone marrow, reducing the number of erythroid precursor cells (Coe et al., 2014), and its intact form (iFGF23) acts on FGFR1 on erythroid precursors, inducing mitochondrial dysfunction and differentiation blockade, collectively suppressing erythroid proliferation and maturation (Park et al., 2024; Courbon et al., 2026). On the other hand, iFGF23 aggravates renal interstitial fibrosis, reduces EPO synthesis, and decreases EPOR expression on erythroid cells, leading to EPO/EPOR pathway impairment (Wang et al., 2025; Coe et al., 2014). Furthermore, FGF23 promotes the release of the inflammatory cytokine IL-6, interferes with iron metabolism, induces hepatic hepcidin expression leading to functional iron deficiency, and enhances splenic phagocytosis of erythrocytes, thereby exacerbating anemia (Park et al., 2024). At the same time, FGF23 accelerates the decline in levels of its co-receptor Klotho, further disrupting hematopoietic homeostasis (Vadakke et al., 2014).

Multiple components of CKD-MBD synergistically disrupt hematopoietic homeostasis. As CKD progresses, iFGF23 continues to rise, and exogenous EPO further elevates FGF23 expression while suppressing Klotho (Toro et al., 2018). This vicious cycle ultimately leads to the development of hyperphosphatemia, hypocalcemia, low 1,25-dihydroxyvitamin D_3_ (1.25(OH)_2_D_3_), elevated parathyroid hormone (PTH), and secondary hyperparathyroidism (Hu et al., 2022; Moe et al., 2006; Carullo et al., 2024; Simic and Babitt, 2021). Existing evidence indicates that these abnormalities are also independent risk factors for aggravating EPO resistance.

Clinical cross-sectional studies have confirmed that hyperphosphatemia is directly associated with anemia (Tran et al., 2016). High-turnover bone disease driven by secondary hyperparathyroidism results in the replacement of hematopoietic tissue in the bone marrow by fibrous tissue, disrupting the hematopoietic microenvironment (Drüeke and Massy, 2016; Gallieni et al., 2000), leading to a reduction in EBI macrophages within the bone marrow microenvironment, failure of HSC retention in the bone marrow, and aberrant mobilization into the peripheral blood (Aleksinskaya et al., 2018). At the same time, a hypocalcemic environment leads to insufficient activation of CaR signaling on HSC surfaces, weakening HSC adhesion, chemotaxis, and homing capacity, and accelerating HSC exhaustion (Lam et al., 2011). In addition, low 1.25(OH)_2_D_3_ increases the expression of inflammatory cytokines in the bone marrow microenvironment (Icardi et al., 2013) and downregulates EPOR expression on erythroid precursors (Alon et al., 2002), while elevated PTH directly impairs the viability of erythroid precursors and erythrocytes, reducing erythrocyte quality (Brancaccio et al., 2004).

Of note, ESAs have limited efficacy in reversing CKD-MBD-induced hematopoietic abnormalities. Although whether EPO directly stimulates osteocyte secretion of FGF23 remains to be elucidated, it is clear that exogenous EPO further elevates FGF23 levels (Toro et al., 2018). Dynamic monitoring and treatment of CKD-MBD are essential to alleviating EPO resistance and enhancing the therapeutic efficacy of renal anemia management.

### 4.4 Accumulation of uremic toxins

In CKD patients, impaired renal function leads to a marked decline in the ability to clear uremic toxins. This, combined with disordered protein anabolic metabolism and abnormal protein fermentation caused by gut microbiota dysbiosis under CKD conditions, results in the accumulation of large amounts of indole, phenol, furan, and amine class uremic toxins that cannot be normally metabolized and excreted, continuously building up in the body (Rysz et al., 2021).

IS, the most representative uremic toxin, interferes with the biological function of EPO through multiple mechanisms. IS downregulates the expression of multiple erythroid-related genes, including *GATA1, EPOR, and β-globin*, blocks erythroid differentiation at the BFU-E stage, and accelerates apoptosis of erythroid precursor cells (Hamza et al., 2023). More refined mechanistic studies have revealed that IS induces overexpression of miR-223 in erythroid precursor cells, reducing the level of the erythroid differentiation factor LMO2 and inhibiting erythroid differentiation (Brisot et al., 2025a). Concurrently, IS upregulates hepatic hepcidin expression through an aryl hydrocarbon receptor (AhR)-dependent pathway and oxidative stress mechanisms, exacerbating functional iron deficiency (Lu et al., 2021; Hamano et al., 2018). Furthermore, IS transported into erythrocytes via organic anion transporter 2 (OAT2), activates NADPH oxidase, mediates oxidative stress, and promotes erythrocyte apoptosis (Lu et al., 2021; Dias et al., 2018).

Other types of uremic toxins in CKD patients also aggravate EPO resistance. For example, 3-carboxy-4-methyl-5-propyl-2-furanpropionate (CMPF) activates the PIEZO1 channel on the erythrocyte membrane, inducing massive calcium influx, disrupting erythrocyte membrane integrity, increasing erythrocyte fragility, and significantly shortening erythrocyte lifespan (Van Spitzenbergen et al., 2025). In addition, cyanate, a byproduct of urea decomposition, modifies the free amino groups of EPO protein, leading to EPO carbamylation, which hinders EPO binding to EPOR and reduces EPO biological activity (Jin, 2013).

### 4.5 Malnutrition

CKD patients often experience long-term gastrointestinal dysfunction and chronic wasting, making them prone to varying degrees of protein-energy malnutrition, which is an independent risk factor for poor response to EPO therapy (Feret et al., 2022; Borelli et al., 2007). Clinical studies have found that patients with EPO resistance are more likely to present with malnutrition-related features, including low serum albumin, low body mass index, and low leptin levels (Feret et al., 2022; Tran et al., 2016; Nikolovski et al., 2024). Mechanistic studies have further revealed the inhibitory effects of malnutrition on erythroid proliferation and differentiation.

Inadequate protein intake and negative nitrogen balance directly induce G1 phase cell cycle arrest in erythroid precursor cells, suppressing their entry into the proliferative cycle, while also reducing the secretion of hematopoietic factors such as SCF(Hastreiter et al., 2020; Borelli et al., 2007). In addition, the low-fat state in CKD is detrimental to erythropoiesis. Under physiological stress and hypoxic conditions, bone marrow adipocytes can release fatty acids to promote oxidative metabolism and provide ATP for HSC and erythroid precursor differentiation; this process is correspondingly attenuated when adipocytes are reduced (Zhang et al., 2023b).

Additionally, deficiencies in folate and vitamin B_12_ impair DNA synthesis in erythroid precursors, resulting in asynchronous nuclear maturation—a recognised contributor to EPO resistance in CKD that warrants clinical recognition and appropriate correction (Improving Global Outcomes Anemia Work Group, 2026).

### 4.6 Other CKD-Related factors contributing to secondary EPO resistance

#### 4.6.1 Gut microbiota dysbiosis

Gut microbiota dysbiosis plays an upstream initiating role in uremic toxin production and nutritional status regulation in CKD patients. In the CKD state, short-chain fatty acid-producing bacteria such as *Faecalibacterium*, *Citrobacter*, *Bifidobacterium*, and *Bacteroides* are decreased in the gut, whereas proteolytic bacteria are relatively increased, leading to increased production of toxins such as IS and worsened nutritional status, collectively aggravating EPO hyporesponsiveness (Zhu et al., 2022). Restoring gut microecological dysbiosis may represent a potential therapeutic strategy to improve ESA responsiveness (Zhu et al., 2022; Rysz et al., 2021).

#### 4.6.2 Interference by angiotensin-converting enzyme inhibitors

Angiotensin-converting enzyme inhibitors (ACEi) are first-line antihypertensive agents in CKD, but they carry a risk of inducing EPO resistance. The mechanism is related to abnormal metabolism of the hematopoietic inhibitory tetrapeptide N-acetyl-seryl-aspartyl-lysyl-proline (Ac-SDKP). Ac-SDKP is primarily degraded and cleared by ACE. By inhibiting ACE activity, ACEi significantly elevate Ac-SDKP levels, which in turn inhibit erythroid precursor entry into the S phase, reduce erythrocyte proliferation and differentiation, and aggravate EPO resistance (Azizi et al., 1996; van der Meer et al., 2005). Although ACEi may reduce erythropoietic responsiveness in some cases, their renoprotective and cardiovascular benefits remain central to CKD management. The use of ACEi should be evaluated in the context of the overall condition of renal anemia.

The roles of the aforementioned external pathological stimuli in EPO resistance are depicted in Figure 3. Moreover, these stimuli can disrupt erythropoiesis via changes in intracellular epigenetic and transcriptional regulation, with lncRNAs serving as a critical molecular network that transduces pathological signals and interferes with erythroid development (Brisot et al., 2025b).

**FIGURE 3:**
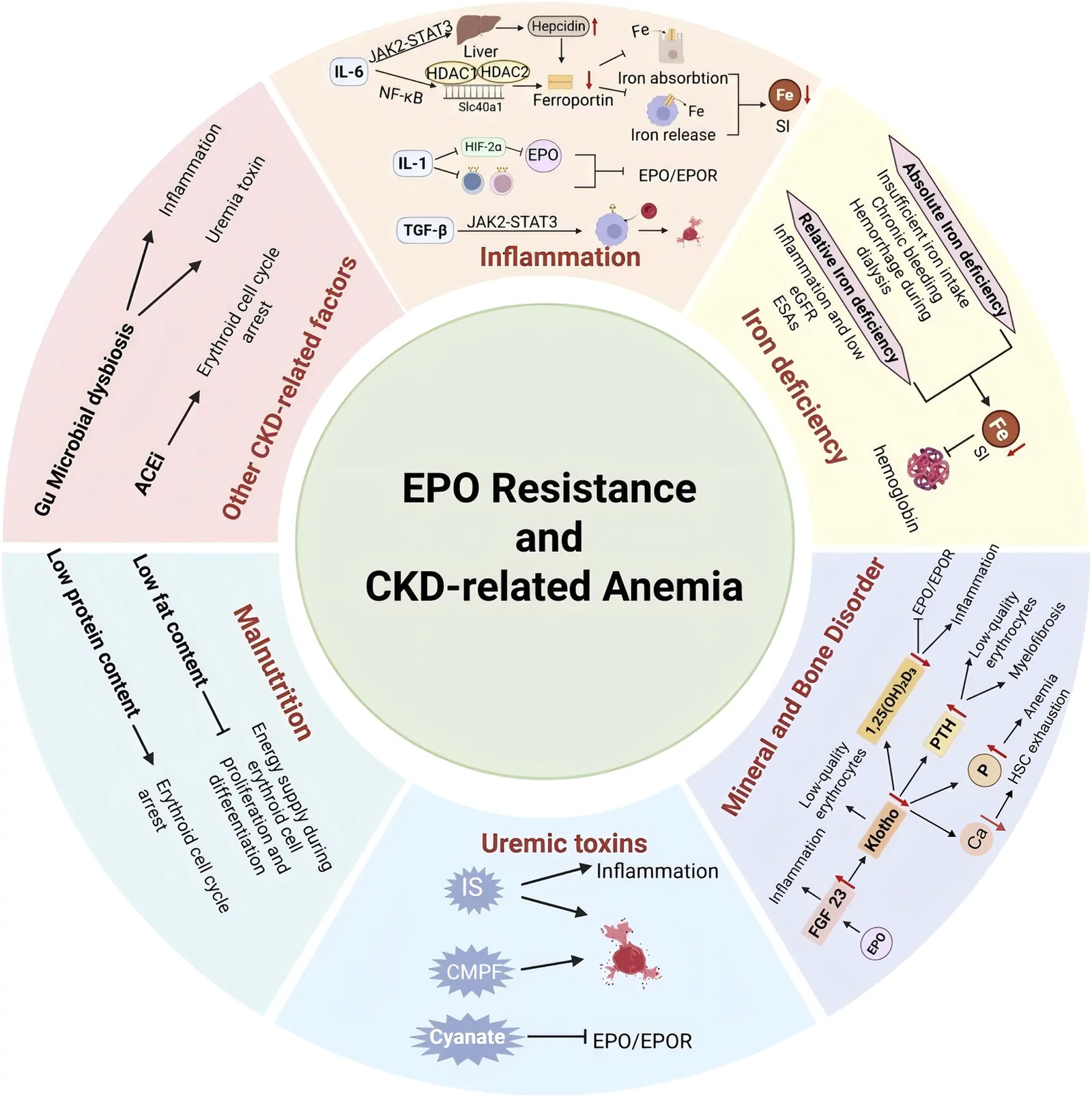
Multiple pathological factors in CKD induce EPO resistance and exacerbate renal anemia. The inflammatory microenvironment, iron deficiency, mineral and bone metabolism disorders, uremic toxins, malnutrition, and other CKD-related factors (such as gut microbiota dysbiosis and ACE inhibitors) contribute to EPO resistance through multiple dimensions, including EPO/EPOR signaling, supply of hematopoietic substrates, and erythrocyte survival, thereby forming a complex pathological network. CKD, chronic kidney disease; EPO, erythropoietin; IL, Interleukin; TGF-β, transforming growth factor-β; HDAC, histone deacetylase; SI, serum iron; ESAs, erythropoiesis-stimulating agents; eGFR, estimating glomerular filtration rate; FGF23, fibroblast growth factor-23 (1.25(OH)2D3, 1,25-dihydroxyvitamin D3; PTH, parathyroid hormone; P, serum phosphorus; Ca, serum calcium; HSC, hematopoietic stem cell; IS, indoxyl sulfate; CMPF, 3-carboxy-4-methyl-5-propyl-2-furanpropionate; ACEi, angiotensin-converting enzyme inhibitosr.

### 4.7 Regulation of erythropoiesis and EPO resistance by long non-coding RNAs

LncRNAs are RNA molecules longer than 200 nucleotides that lack protein-coding capacity. They are dynamically expressed throughout erythroid maturation and can be classified, based on their biological effects, into erythropoiesis-promoting and erythropoiesis-suppressing subtypes. Through epigenetic mechanisms such as chromatin remodelling, miRNA sponging, and alternative splicing of pre-mRNA, lncRNAs cooperate with miRNAs to orchestrate haematopoietic lineage commitment, apoptosis, haem synthesis, and the enucleation and terminal maturation of late normoblasts (Brisot et al., 2025b).

Under pathological conditions where iron metabolism disorders coexist with chronic inflammation, dysregulation of the lncRNA–mRNA co-expression network has been demonstrated to contribute to anemia (Lin et al., 2025). Studies in kidney injury have shown that lncRNAs NEAT1, MALAT1, and HOTAIR can upregulate inflammatory pathways such as NF-κB, IL-6, and TNF-α, thereby promoting renal interstitial fibrosis and worsening renal function. The resulting inflammatory microenvironment further constitutes an important pathological trigger for EPO resistance; however, direct regulation of *Epo* transcription in REPCs by lncRNAs remains to be validated (Puri and Gaikwad, 2026).

In addition, the CKD pathological milieu induces alterations in lncRNA expression profiles that impair erythropoiesis. For instance, under microinflammatory conditions, NF-κB suppresses lncRNA Saf expression, which in turn inhibits Saf-mediated alternative splicing of Fas pre-mRNA, leading to Fas-induced apoptosis of erythroid cells (Brisot et al., 2025b). NEAT1 binds erythroid transcription factors such as GATA1; knockdown of NEAT1 in human umbilical cord blood CD34^+^ haematopoietic progenitor cells suppresses erythropoiesis, although the precise mechanism by which NEAT1 regulates erythroid differentiation remains to be elucidated (Brisot et al., 2025b). In CKD patients with comorbid diabetes, despite lower hemoglobin levels, both serum and urinary NEAT1 levels are higher than those in healthy controls (Petrica et al., 2021). Consistent with the above observations, most current studies consider NEAT1 a detrimental factor in CKD-associated inflammation and fibrosis, and the specific role of NEAT1 in renal anemia warrants further investigation (Abd-Elmawla et al., 2024). In CKD, lncRNA H19 levels increase with advancing renal dysfunction and correlate positively with serum inflammatory cytokines (IL-6, TNF-α), ferritin, phosphorus, and PTH (Okuyan et al., 2021). Abnormal H19 expression has also been observed in transfusion-dependent β-thalassaemia patients, and its expression is significantly associated with iron-regulatory molecules HAMP and FAM132B, further suggesting that H19 participates in the regulation of iron metabolism and hemoglobin synthesis (Al-Sanabra et al., 2025). lncRNA UCA1 is involved in haem biosynthesis; its deficiency arrests erythroid differentiation at the Pro-E stage. *In vitro* experiments have shown that cisplatin downregulates UCA1 expression in renal tubular epithelial cells; given the phenotype of cisplatin-induced renal anemia, it is plausible that UCA1 downregulation contributes to the pathogenesis of this anemia (Lugones et al., 2025; Estrela et al., 2021).

Serum lncRNA KCNQ1OT1 is significantly upregulated in CKD patients, and its predicted downstream target miR-9 is independently associated with the ERI in hemodialysis patients, suggesting that the KCNQ1OT1/miR-9 regulatory axis may be involved in EPO resistance (El Samaloty et al., 2026). Although peripheral blood lncRNAs show potential for stratifying EPO resistance (Zhou et al., 2025), existing mechanistic studies on lncRNAs are largely based on cellular and animal models, while current clinical trials only provide correlational evidence. There is a lack of large-scale clinical transcriptomic data in CKD to support these findings, and the underlying mechanisms remain to be explored. No targeted clinical agents are yet available, and the translational gap to clinical application remains substantial.

In summary, the various systemic peripheral pathological triggers in CKD, together with lncRNA-mediated epitranscriptional dysregulation, synergistically constitute a complex regulatory network of EPO resistance. This network represents the core pathological basis underlying the inadequate therapeutic response to ESAs and HIF-PHIs. Given this multi-pathway pathogenic background, clinical management should follow a diagnostic and therapeutic algorithm that prioritises the screening and correction of reversible resistance factors, followed by individualised selection of anti-anemia medications.

## 5 Pharmacological treatment of renal anemia

Before initiating ESAs, HIF-PHIs, or up-titrating their dosages, it is imperative to systematically identify and correct all reversible triggers of EPO resistance, so as to avoid the additional safety risks, including cardiovascular and thrombotic events associated with merely increasing the dose of ESAs and HIF-PHIs. In light of the multipathogenic mechanisms discussed above, we have summarised in Table 1 the major reversible triggers of EPO resistance, the corresponding screening parameters, and the recommended corrective strategies. Clinical management should adopt a stepwise approach that prioritises trigger screening, targeted correction, and optimisation of the type and dosage of anemia medications. The clinical application of each class of agents described in the subsequent sections is predicated on this preliminary assessment process.

**TABLE 1:** Major triggers of EPO resistance in renal anemia: diagnostic assessment and corrective strategies.

Reversible triggers	Key diagnostic assessment parameters	Corrective strategies
Iron deficiency	Serum iron, ferritin, transferrin saturation, total iron-binding capacity, hepcidin (Improving Global Outcomes Anemia Work Group, 2026)	Prefer oral iron for absolute deficiency; prefer intravenous iron for functional or severe deficiency (Bazeley and Wish, 2022; Chukwu et al., 2023)
Infection or chronic inflammation	Inflammatory cytokines (IL-1, IL-6, TGF-β1, CRP, etc.); screen for occult infections (vascular access, dental, pulmonary, etc.) (Gluba-Brzózka et al., 2020)	Anti-infective therapy; control sterile inflammation; refractory cases may consider anti-IL-6 agents (investigational) (Neuen et al., 2025)
Overt or occult blood loss	Fecal occult blood test, gastrointestinal endoscopy; dialyzer clotting and residual blood loss (Bazeley and Wish, 2022)	Identify and control bleeding source; optimise anticoagulation regimen for dialysis
CKD-MBD	FGF23, PTH, serum calcium, phosphorus, 1.25(OH)2D3 (Hu et al., 2022; Simic and Babitt, 2021)	Active vitamin D analogues, calcimimetics; parathyroidectomy if refractory to medical therapy (Weng et al., 2025; Xu et al., 2025; Lau et al., 2018)
Accumulation of uremic toxins/inadequate dialysis	Kt/V; blood urea nitrogen; residual renal function; evaluate uremic clinical features together with dialysis adequacy (National Kidney Foundation, 2015)	Optimise dialysis prescription (frequency, duration, blood flow rate and dialyzer membrane type); enhancement of uraemic toxin clearance (National Kidney Foundation, 2015)
Nutritional deficiencies	Serum albumin, body mass index, folate, vitamin B12 (Feret et al., 2022; Tran et al., 2016; Nikolovski et al., 2024; Improving Global Outcomes Anemia Work Group, 2026)	Supplement protein-energy; supplement folate and vitamin B12 as needed (Improving Global Outcomes Anemia Work Group, 2026)
Drug interference (ACEi)	Review of medication history (Santos et al., 2020)	Re-evaluate indications; weigh renoprotective benefit against exacerbation of anemia; switch to alternative antihypertensives when clinically feasible
Concomitant haematologic disorders	Peripheral blood smear, bone marrow examination (if indicated); anti-ESA antibody testing (Improving Global Outcomes Anemia Work Group, 2026)	Consult haematology; if pure red cell aplasia is confirmed, discontinue ESAs and initiate immunosuppressive therapy (Improving Global Outcomes Anemia Work Group, 2026)

IL, interleukin; TGF-β1, transforming growth factor-β1; CRP, C-reactive protein; CKD-MBD, chronic kidney disease-mineral and bone disorders; FGF23, fibroblast growth factor 23; PTH, parathyroid hormone; (1.25(OH)_2_D_3_, 1,25-dihydroxyvitamin D3; Kt/V, urea fractional clearance index; ACEi, angiotensin-converting enzyme inhibitors; ESAs, erythropoiesis-stimulating agents.

Specifically, the pre-treatment evaluation can be summarised as a sequential approach: “confirm anemia and iron status—systematically screen for and correct reversible triggers—reassess hemoglobin and ESA responsiveness—then select or adjust erythropoiesis-stimulating agents.” The first step is to assess absolute or functional iron deficiency; subsequently, screen for active infection or chronic inflammation, overt or occult blood loss, CKD-MBD, uraemic toxin accumulation/inadequate dialysis, protein-energy malnutrition, and folate or vitamin B_12_ deficiency, while also reviewing medications that may interfere with erythropoiesis, and exclude concomitant haematological disorders when the response is abnormal or blood counts are atypical. Only after these reversible factors have been addressed should ESAs, HIF-PHIs, and adjunctive therapies be individually initiated or adjusted based on hemoglobin levels, ERI, CKD stage, dialysis status, and cardiovascular risk (Improving Global Outcomes Anemia Work Group, 2026; Gluba-Brzózka et al., 2020; Bazeley and Wish, 2022; National Kidney Foundation, 2015; Feret et al., 2022; Borelli et al., 2007; Santos et al., 2020).

Based on the dual pathological core of renal anemia—insufficient EPO synthesis and EPO resistance—current pharmacological agents can be categorized into three major classes: classic replacement drugs, adjunctive drugs that ameliorate EPO resistance, and novel agents with dual actions. For EPO deficiency, classic ESAs effectively compensate for the insufficiency and remain the mainstream therapy. HIF-PHIs have the advantage of promoting endogenous EPO synthesis while improving EPO resistance, and have been approved for the treatment of renal anemia in multiple countries (Haase et al., 2024). Iron agents are the most widely used adjunctive therapy to alleviate EPO resistance, forming the cornerstone of renal anemia treatment together with ESAs and HIF-PHIs. In addition, sodium-glucose cotransporter 2 inhibitors (SGLT-2i), originally developed for glycemic control in diabetic patients, have been shown not only to have renoprotective effects—reducing proteinuria and slowing the decline in eGFR—but also, as growing evidence suggests, to be useful in treating renal anemia (Koshino et al., 2023a). Traditional Chinese medicine therapies have also demonstrated positive effects in CKD management (Li et al., 2020a), with potential mechanisms including delaying renal fibrosis, elevating EPO levels, and alleviating EPO resistance. Although a comprehensive array of anti-anemia agents is currently available for renal anemia, each has its own limitations. Table 2 briefly compares these therapeutic options, and the following sections will review the current status and bottlenecks in their clinical application.

**TABLE 2:** Brief comparison of therapeutic agents for renal anemia.

Drug category	Representative drugs	Key pharmacokinetic features	Main advantages	Key limitations
Short-acting ESAs	Epoetin alfa, Epoetin beta	t1/2 6–8 h, administered 2 to 3 times weekly (Sinclair, 2013)	Small Hb fluctuation, easily adjustable dosing (Ku et al., 2023)	Frequent injections; poor adherence (Ku et al., 2023)
Long-acting ESAs	Darbepoetin alfa	t1/2 > 25 h, once weekly (Sinclair, 2013)	Reduced dosing frequency (Sinclair, 2013)	High Hb variability; inconsistent findings from long-term cardiovascular studies (Kang and Kim, 2025; Desai et al., 2024; Sakaguchi et al., 2019)
	Pegmolesatide	t1/2 70 h, once monthly (Ma et al., 2025)	Long-acting erythropoiesis; phase III trial in China showed non-inferiority to short-acting ESAs(Zhang et al., 2023a)	Increased mild infections and hypotension adverse events (Zhang et al., 2023a)
	CERA	t1/2 130 h, once every 2–4 weeks (Macdougall and Eckardt, 2006)	stable erythropoiesis (Macdougall and Eckardt, 2006)	Relatively high Hb variability; insufficient evidence for hard endpoints (Kang and Kim, 2025)
Oral iron agents	Ferrous sulfate, ferric citrate	Orally daily (Bazeley and Wish, 2022)	Convenient and safe (Bazeley and Wish, 2022),ferric citrate effectively improves iron metabolism and lowers serum FGF23 levels (Block et al., 2015; Womack et al., 2020)	Absorption inhibited by hepcidin; gastrointestinal adverse effects common (Bazeley and Wish, 2022)
Intravenous iron agents	Iron sucrose, iron dextran, ferric carboxymaltose	Intermittent infusion (Bazeley and Wish, 2022)	Rapid replenishment of functional iron stores (Bazeley and Wish, 2022)	Risks of allergy, oxidative stress, and infection (Bazeley and Wish, 2022)
HIF-PHIs	Roxadustat	Orally 3 times weekly; non-selective PHD inhibition (Thévenod et al., 2022)	Significant Hb elevation (Chen et al., 2023, Nasiri et al., 2025)	Higher risks of thrombosis and vascular access complications (Chen et al., 2023; Nasiri et al., 2025); risk of Hb overshoot (Tamaki et al., 2025)
	Daprodustat, Vadadustat	Orally daily; preferentially targets PHD3[1[166]70]	Daprodustat: pronounced improvement in iron metabolism and fewer vascular access complications (Nasiri et al., 2025); Vadadustat: relatively lower risk of hypertension (Chen et al., 2023)	Daprodustat: gastrointestinal adverse effects common (Nasiri et al., 2025); Vadadustat: Hb correction effect weaker than ESAs(Chen et al., 2023)
	Molidustat	Orally daily; mainly targets PHD2 (Gaete et al., 2021)	Relatively lower risk of hyperkalaemia (Nasiri et al., 2025)	Potential safety concerns due to HIF activation, including tumours, diabetic retinopathy, and cyst progression (Ku et al., 2023)
	Enarodustat	Orally daily; preferentially targets PHD1 (Wu et al., 2026)	Greater selectivity for renal tissue; improves iron metabolism and stably maintains Hb target (Wu et al., 2026)	Higher risk of gastrointestinal side effects (Chen et al., 2023)
Targeting inflammation-hepcidin pathway	DISC-0974	Intravenous; blocks BMP-SMAD pathway and inhibits HAMP transcription (Gangat and Tefferi, 2025)	Suppresses hepcidin synthesis at source, relieves iron-estricted erythropoiesis (Gangat and Tefferi, 2025)	Phase Ib trial data not yet published; overall clinical benefit unknown (ClinicalTrials.gov, NCT05745883)
​	PRS-080, Lexaptepid	Intravenous; directly binds and neutralises circulating free hepcidin (Hohlbaum et al., 2018; van Eijk et al., 2014)	Directly inactivates circulating hepcidin, increases serum iron and transferrin saturation (Hohlbaum et al., 2018; van Eijk et al., 2014)	Phase II trials did not meet prespecified efficacy endpoints; clinical development discontinued (ClinicalTrials.gov, NCT03325621, NCT02079896)
	Ziltivekimab, Clazakizumab	Subcutaneous injection	Targets IL-6, suppresses inflammation and improves iron utilization; Ziltivekimab reduces the ERI and ESAs requirements in ESA-hyporesponsive dialysis patients, and improves Hb in non-dialysis CKD patients (Pergola et al., 2021; Pergola et al., 2024)	Remains under investigation; Clazakizumab has not been shown to clearly increase Hb levels in the dialysis population (Neuen et al., 2025), and a phase III trial is currently ongoing (ClinicalTrials.gov, NCT05485961)
	nanosized iron oxide nanoparticles	Orally	Dual effect of iron supplementation and anti-inflammation; effectively corrects anemia in CKD mice (Hsiao et al., 2023)	Not yet clinically applied (Hsiao et al., 2023)
CKD-MBD targeted adjunctive agents	Cinacalcet, Vitamin D	Orally	Improve calcium-phosphorus metabolism and reduce ERI (Weng et al., 2025; Xu et al., 2025)	Limited Hb-elevating effect alone; caution needed for hypercalcaemia and hyperphosphataemia
Other adjunctive agents	SGLT-2i, TCM	Orally	SGLT-2i: post hoc analyses of large RCTs confirmed improvement in iron utilisation and Hb elevation (Koshino et al., 2025; Oshima et al., 2020; Koshino et al., 2023b); TCM: may synergistically improve EPO resistance via multiple pathways (Wang et al., 2020; Li et al., 2023)	Neither has a clear indication specifically for renal anemia; TCM lacks large-scale evidence

CERA, continuous erythropoietin receptor activator; Hb, hemoglobin; ESAs, erythropoiesis-stimulating agents; FGF23, fibroblast growth factor 23; HIF-PHIs, hypoxia-inducible factor prolyl hydroxylase inhibitors; PHD, prolyl hydroxylase domain; IL-6, interleukin-6; CKD-MBD, chronic kidney disease-mineral and bone disorders; EPO, erythropoietin; ERI, EPO, resistance Index; SGLT-2i, sodium-glucose cotransporter 2 Inhibitors; RCT, randomized controlled trial; TCM, traditional Chinese medicine.

### 5.1 Erythropoiesis-stimulating agents

ESAs are exogenous EPO mimetics and represent the first-line foundational therapy for renal anemia. They are classified into short-acting and long-acting formulations, which differ in molecular modification, pharmacokinetics, and clinical characteristics (Palmer et al., 2014; Improving Global Outcomes Anemia Work Group, 2026). The erythropoietic efficacy of ESAs exhibits a clear dose–response relationship, and hemoglobin target management is the core principle of individualized clinical dosing.

#### 5.1.1 Pharmacological classification and efficacy evaluation

Epoetin alfa and epoetin beta are representative short-acting ESAs. They have a low degree of glycosylation and a short *in vivo* half-life of only 6–8 h, requiring subcutaneous or intravenous injection 2–3 times per week (Sinclair, 2013). These agents provide a steady onset of action and are easily dose-adjustable, making them more suitable for renal anemia patients with significant hemoglobin fluctuations. Although regular administration improves monitoring, it also increases the burden on both healthcare providers and patients, reducing treatment adherence (Ku et al., 2023).

Long-acting ESAs, including darbepoetin alfa, pegmolesatide and continuous erythropoietin receptor activator (CERA), substantially reduce injection frequency and improve adherence. Darbepoetin alfa, which contains five additional N-glycosylation sites, has a prolonged half-life of over 25 h (Sinclair, 2013). Pegmolesatide is an EPO-mimetic peptide composed of natural amino acids that specifically binds to EPOR, activating the JAK2/STAT5 and MAPK/ERK signaling cascades to promote erythrocyte proliferation and differentiation. In addition, PEGylation extends its half-life to approximately 70 h. Hematopoietic cells pretreated with pegmolesatide exhibit a greater and more sustained compensatory upregulation of EPOR expression after drug withdrawal, maintaining cellular sensitivity to the ligand and ultimately achieving a long-acting erythropoietic effect far superior to that of conventional ESAs, with a clinical dosing regimen of once monthly (Ma et al., 2025). CERA is generated by covalently conjugating a methoxy polyethylene glycol side chain to the epoetin beta molecule, resulting in a molecular weight approximately twice that of epoetin beta. It has a half-life of up to 130 h and exhibits slow dissociation from the EPOR, thereby enabling sustained and stable stimulation of erythropoiesis (Macdougall and Eckardt, 2006). Therefore, based on these pharmacological features, CERA should be classified as a long-acting ESA, rather than being considered as a therapeutic category independent of ESAs. Its core characteristics include sustained activation of the EPOR, a prolonged elimination half-life, and a lower dosing frequency. Although the various ESAs described above differ in molecular structure and pharmacokinetic properties, they are all exogenous EPO mimetics and share common clinical limitations, including ESA hyporesponsiveness and cardiovascular safety concerns.

#### 5.1.2 Limitations in application

Currently, there is no universally accepted definition for ESA hyporesponsiveness, and different criteria have been proposed by the National Institute for Health and Care Excellence (NICE), the Kidney Disease: Improving Global Outcomes (KDIGO) guidelines, clinical practice algorithms (CPA), and the ERI. Depending on the definition used, the reported prevalence ranges from 5.2% to 47.6%. This wide variation not only reflects the complexity of this clinical issue but also poses substantial challenges to diagnostic consistency and cross-study comparisons of research findings (Atzinger et al., 2025). Nevertheless, it is clear that when the aforementioned synergistic factors of EPO resistance are present, the efficacy of ESAs is compromised (Zheng et al., 2023). Epidemiological evidence indicates that ESA hyporesponsiveness is not only a decisive factor for poor anemia treatment response (Zheng et al., 2023) but also an independent risk factor for all-cause mortality, cardiovascular events, and disease progression in CKD patients (Zhao et al., 2024).

Cardiovascular risk is a major safety concern associated with ESAs. Whether short-acting and long-acting ESAs differ in long-term patient outcomes remains unclear. A retrospective study from South Korea compared hemoglobin variability and hard clinical endpoints among short-acting ESAs, darbepoetin alfa, and CERA in hemodialysis patients. The results showed that hemoglobin variability was significantly lower with short-acting ESAs than with long-acting agents. The authors suggested that this finding might be influenced by the Korean health insurance policy, which mandates strict maintenance of hemoglobin levels within target ranges. Meanwhile, the study observed a marginal survival benefit with long-acting ESAs, but did not detect an independent association between hemoglobin variability and all-cause mortality or cardiovascular events (Kang and Kim, 2025). In addition, a study involving nearly 70,000 hemodialysis patients in the United States found that darbepoetin alfa showed no significant difference in cardiovascular risk compared with short-acting ESAs, but was associated with a 9% relative reduction in all-cause mortality (Desai et al., 2024). However, a Japanese study reported that although the longer half-life and greater bioactivity of darbepoetin alfa better maintained hemoglobin levels, it was associated with a 13% increase in mortality among hemodialysis patients, and this elevated risk was related to higher doses (Sakaguchi et al., 2019). For pegmolesatide, a Phase III clinical trial in Chinese dialysis patients demonstrated non-inferiority to short-acting ESAs, with similar hemoglobin fluctuation amplitudes between the two agents, no statistically significant differences in composite cardiovascular endpoints or all-cause mortality, and largely overlapping adverse event profiles—with hypertension being the most common. However, pegmolesatide showed higher incidences of adverse events such as hypotension and upper respiratory tract infections (Zhang et al., 2023a). The divergent findings on long-acting ESAs suggest that their effects on patient mortality outcomes may be confounded by multiple factors, including population characteristics, regional treatment protocols, and dosing. Further high-quality clinical studies with larger sample sizes and long-term follow-up are needed to confirm the long-term benefits and safety of different long-acting ESA formulations.

In addition, a very small proportion of patients may develop anti-ESA antibodies, leading to pure red cell aplasia. In such cases, immediate drug discontinuation and initiation of immunosuppressive therapy are required (Ma et al., 2024).

### 5.2 Iron agents

Iron deficiency is common in patients with renal anemia, and iron supplementation significantly improves the efficiency of erythropoiesis (Wang et al., 2023; Ogolla et al., 2025). Clinically, oral iron agents are generally preferred for patients with absolute iron deficiency; for those with severe iron deficiency, intravenous iron is directly recommended (Bazeley and Wish, 2022). According to the 2026 KDIGO guideline recommendations: for CKD stage G5 patients, intravenous iron is preferred when ferritin ≤500 ng/mL and transferrin saturation (TSAT) ≤30%; for non-dialysis or peritoneal dialysis patients, treatment is initiated when ferritin <100 ng/mL with TSAT <40%, or when 100 ≤ ferritin <300 ng/mL with TSAT <25%. During treatment, routine iron administration should be suspended if ferritin >700 ng/mL or TSAT ≥40% (Wang et al., 2023; Improving Global Outcomes Anemia Work Group, 2026).

#### 5.2.1 Pharmacological classification and efficacy evaluation

Oral iron agents include ferrous sulfate and ferric citrate. They offer the advantages of convenient use and high safety, making them suitable for non-dialysis patients and those with mild-to-moderate iron deficiency. However, their absorption is significantly influenced by intestinal pH, food intake, and hepcidin levels, and gastrointestinal adverse effects such as nausea, constipation, and diarrhea are common, limiting long-term use in some patients. Intravenous iron agents include iron sucrose, iron dextran, and ferric carboxymaltose (FCM). These bypass intestinal absorption regulation and rapidly replenish functional iron stores, making them particularly suitable for dialysis patients, those with severe anemia, and those intolerant to oral iron. However, they may induce oxidative stress, allergic reactions, and infection risks, requiring strict adherence to indications and dosing (Bazeley and Wish, 2022).

Oral iron agents not only improve iron metabolism but also reduce serum FGF23 levels, with ferric citrate showing superior effects over ferrous sulfate in this regard. Given that FGF23 is a core molecule in CKD-MBD and EPO resistance, this property confers on iron agents a broader pathological intervention significance beyond simple iron supplementation (Block et al., 2015; Womack et al., 2020). However, a randomized controlled trial (RCT) showed that although ferric citrate was superior to ferrous sulfate in improving iron metabolism and iFGF23 levels, there was no significant difference in hemoglobin levels between the two groups, suggesting that clinical efficacy improvements need to be considered in conjunction with dose adjustments of ESAs/HIF-PHIs(Womack et al., 2020).

Clinical evidence indicates that intravenous iron agents are generally superior to oral iron agents in improving anemia and iron metabolism parameters, and this advantage is significantly modified by inflammation and the type of iron deficiency (Chukwu et al., 2023). In a clinical trial involving 150 renal anemia patients from rural India, iron sucrose was more effective than ferrous sulfate in raising hemoglobin and improving ferritin levels (Agrawal et al., 2022). FCM more significantly improved anemia and iron metabolism parameters compared with ferrous sulfate, and also reduced ESA requirements, suggesting a potential advantage in alleviating EPO resistance (Cirillo et al., 2021). However, functional iron deficiency significantly attenuates its efficacy (Chukwu et al., 2023).

### 5.3 Hypoxia-inducible factor prolyl hydroxylase inhibitors (HIF-PHIs)

HIF-PHIs represent a breakthrough class of agents in the field of renal anemia over the past decade. By competitively inhibiting PHD1/2/3, they simultaneously promote endogenous EPO synthesis and alleviate EPO resistance. To date, multiple agents in this class have been approved globally, including roxadustat, daprodustat, vadadustat, molidustat, and enarodustat (Akizawa et al., 2020a; Zhou et al., 2023; Sugahara et al., 2022).

Although HIF-PHIs have a slower onset of hemoglobin elevation compared with ESAs, multiple Phase III clinical trials have confirmed that their efficacy in raising hemoglobin is non-inferior to that of ESAs. When compared with short-acting ESAs, HIF-PHIs even show a certain advantage in the magnitude of hemoglobin increase. Concurrently, the overall cardiovascular risk of HIF-PHIs is comparable to that of ESAs(Huang et al., 2023; Wish, 2022; Zhou et al., 2023). Most importantly, HIF-PHIs are suitable for renal anemia patients with EPO resistance; their erythropoietic efficacy is independent of inflammatory status (Zheng et al., 2023), and they improve iron metabolism parameters, reducing weekly intravenous iron requirements by 3.1 mg (Minutolo et al., 2024).

#### 5.3.1 Pharmacological classification and efficacy evaluation

Different agents exhibit varying selectivity for PHD isoforms, which directly determines their efficacy and safety profiles. Roxadustat is a non-selective inhibitor of PHD1/2/3 with a broad spectrum of action, administered three times weekly, and significantly reduces hepcidin while improving inflammation and lipid profiles. Daprodustat and vadadustat preferentially target PHD3 and are taken once daily orally, with stable plasma concentrations (Thévenod et al., 2022). Molidustat primarily targets PHD2, while enarodustat tends to preferentially inhibit PHD1; both are orally administered once daily, with the latter showing greater selectivity for renal tissue (Thévenod et al., 2022; Wu et al., 2026; Gaete et al., 2021).

In the treatment of renal anemia, HIF-PHIs are non-inferior to ESAs in correcting anemia and are generally superior to ESAs in improving iron metabolism, although different HIF-PHI subtypes each have their own respective advantages and disadvantages. A 2025 meta-analysis encompassing over 30,000 renal anemia patients showed that roxadustat and daprodustat achieved greater hemoglobin increases than ESAs, with roxadustat being the most effective; however, it was associated with higher rates of thrombosis and vascular access complications (Chen et al., 2023; Nasiri et al., 2025). Daprodustat, on the other hand, demonstrated more pronounced advantages in improving iron metabolism, fewer vascular access complications, but a higher propensity for gastrointestinal reactions (Nasiri et al., 2025). The same study additionally noted that molidustat carries a lower risk of hyperkalemia (Nasiri et al., 2025). Vadadustat was less effective than ESAs in correcting hemoglobin but was associated with a lower risk of hypertension (Chen et al., 2023). The ENARODIAL trial indicated that after switching from ESA therapy to enarodustat in dialysis patients, a higher proportion achieved target hemoglobin levels (79.6% vs. 51.0%), with improved iron metabolism parameters, suggesting that enarodustat is superior to ESAs in maintaining hemoglobin stability (Wu et al., 2026). However, the meta-analysis noted that this agent has a higher incidence of gastrointestinal adverse effects (Chen et al., 2023).

The selection of anemia medications still requires head-to-head trials and real-world studies to provide further reference. In clinical practice, attention should be directed not only to the average hemoglobin response, but also to the interindividual heterogeneity in treatment response and the risk of early hemoglobin overshoot after switching agents—both of which represent clinically relevant issues that cannot be overlooked. A retrospective study of non-dialysis CKD patients with renal showed that after conversion from ESAs to roxadustat, 34.8% of patients developed hemoglobin overshoot (defined as Hb > 12.5 g/dL) within an 8-week observation period. This incidence was significantly higher than that in the control group continuing ESAs (3.2%; OR 20.2, 95% CI 3.13–130). Younger age, higher baseline hemoglobin and hematocrit levels were identified as risk factors for hemoglobin overshoot (Tamaki et al., 2025). Meanwhile, a subgroup analysis of a phase III trial in the hemodialysis population indicated that dialysis patients switched from ESAs to HIF-PHIs also exhibited heterogeneity in response, with those having high baseline high-sensitivity C-reactive protein (hs-CRP) showing a more pronounced hemoglobin increase after conversion (Akizawa et al., 2020b). On one hand, this phenomenon suggests that HIF-PHIs can partially ameliorate inflammation-mediated EPO resistance; on the other hand, it underscores the potential risk of hemoglobin overcorrection even in the switching setting. Therefore, close monitoring of hemoglobin is warranted after switching anemia medications in clinical practice.

It should be emphasised that RCTs and meta-analyses typically assess efficacy based on mean hemoglobin changes and target achievement rates over relatively long observation windows. While these endpoints adequately capture overall treatment effectiveness, they may obscure early individual fluctuations in response after drug switching. Real-world data are well positioned to fill this evidence gap. Available evidence suggests that, following conversion from ESAs to HIF-PHIs, particular attention should be paid to the rate of hemoglobin rise during the first 4–8 weeks, rather than focusing solely on the final mean value. In patients with higher baseline hemoglobin/hematocrit or greater inflammatory burden, more intensive monitoring and timely dose adjustment are warranted to mitigate the risk of excessive hemoglobin elevation and its potential thrombotic consequences (Tamaki et al., 2025; Akizawa et al., 2020b).

#### 5.3.2 Limitations in application

The major safety concerns of HIF-PHIs stem from excessive activation of HIF-1α, which may lead to elevated VEGF expression that could promote tumor progression, exacerbate diabetic retinopathy, and accelerate cyst growth in polycystic kidney disease (Ku et al., 2023). Some patients also experience hyperkalemia and blood pressure fluctuations. Currently, no evidence has confirmed that HIF-PHIs directly induce tumors, but caution is advised in patients at high risk of malignancy (Seeley et al., 2017). Regarding the mitigation of the broad physiological effects induced by HIF-PHIs, combination with HIF-2α agonists may be a viable approach. Studies have shown that co-administration of the HIF-2α agonist amide thiazole (AT-1) with molidustat produces a more potent erythropoietic and EPO-stimulating effect (Chu et al., 2025). Similar observations have been made with the combination of a HIF-2α agonist and daprodustat (Guo et al., 2025). The combination of HIF-2α agonists with HIF-PHIs can specifically amplify EPO synthesis while reducing HIF-1α-mediated side effects, representing a direction for next-generation drug development.

### 5.4 Other potential therapeutic agents

#### 5.4.1 Agents targeting inflammation and hepcidin

Strategies to correct inflammation-mediated EPO resistance in CKD can be broadly classified into two categories: those targeting the hepcidin pathway and those targeting inflammatory pathways. Interventions directed at hepcidin can be achieved by suppressing its biosynthesis or downregulating its expression. DISC-0974 is a monoclonal antibody targeting the BMP co-receptor hemojuvelin (HJV), which blocks BMP-SMAD signalling activation and thereby inhibits HAMP gene transcription, reducing hepcidin production at the source (Gangat and Tefferi, 2025). A phase Ib trial in non-dialysis-dependent renal anemia (ClinicalTrials.gov, NCT05745883) has completed subject enrolment and the intervention phase, although final complete data have not yet been formally published. Representative agents that directly neutralise circulating free hepcidin include PRS-080 (an engineered anticalin-based molecule) and Lexaptepid (a chemically synthesised structured mirror-image oligoribonucleotide), both of which inhibit the biological function of hepcidin (Hohlbaum et al., 2018; van Eijk et al., 2014). A phase I trial of PRS-080 in dialysis patients demonstrated favourable safety and tolerability, with dose-dependent reductions in serum hepcidin and increases in serum iron levels, although no significant improvement in hemoglobin was observed after a single dose (Renders et al., 2019). However, phase II trials of PRS-080 (ClinicalTrials.gov, NCT03325621) and Lexaptepid (ClinicalTrials.gov, NCT02079896) did not meet robust clinical efficacy endpoints, leading to discontinuation of further clinical development.

In addition to agents directly targeting the hepcidin pathway, intervention strategies directed at upstream inflammatory factors also hold clinical value. These two strategies differ pharmacologically: targeting hepcidin mainly alleviates iron-restricted haematopoiesis, whereas upstream anti-inflammatory therapy inhibits the IL-6/JAK-STAT-hepcidin axis and counteracts inflammation-mediated impairment of EPO/EPOR signalling, erythroid differentiation, and red cell survival, making it more appropriate for patients with overt inflammation and ESA resistance (Pergola et al., 2021; Pergola et al., 2024; Neuen et al., 2025). Ziltivekimab and clazakizumab are both monoclonal antibodies targeting IL-6 that block IL-6-mediated inflammatory pathways, representing potential therapeutic options for inflammation-related EPO resistance. In a RCT involving 61 hemodialysis patients, the IL-6 monoclonal antibody ziltivekimab was shown to suppress the expression of inflammatory markers such as CRP, increase iron utilization and serum albumin levels, and reduce ERI (Pergola et al., 2021). The subsequent RESCUE trial, which enrolled 256 patients with CKD stages 3–5 and baseline hs-CRP ≥2 mg/L, showed that ziltivekimab significantly increased hemoglobin levels and improved iron metabolism in this population (Pergola et al., 2024). In contrast, clinical evidence for clazakizumab is derived primarily from the maintenance dialysis population. In dialysis patients with baseline hs-CRP ≥2 mg/L, clazakizumab downregulated inflammatory markers, increased albumin, improved iron availability, and reduced ESAs requirements. However, no statistically significant increase in hemoglobin was observed in that study, which may be attributable to confounding effects from the permissive adjustment of ESAs doses allowed in the trial (Neuen et al., 2025). A larger phase III trial of clazakizumab (ClinicalTrials.gov, NCT05485961) is currently ongoing and will further clarify its effects on anemia, EPO resistance, and hard cardiovascular endpoints in dialysis patients. Overall, the role of IL-6 inhibitors in improving renal anemia remains exploratory, and the current evidence is still limited and should not be overestimated. Notably, the RCT of ziltivekimab in dialysis patients was performed on a stable background of ESAs (including epoetin alfa, darbepoetin alfa, or CERA), and its clinical significance resides not in rapid hemoglobin elevation but in lowering ERI and ESAs requirements while maintaining anemia control (Pergola et al., 2021). Thus, anti-IL-6 therapy should currently be viewed as a sensitising/sparing adjunct for inflammation-driven ESA hyporesponsiveness rather than a replacement for ESAs. Whether a particular ESAs-ziltivekimab combination (e.g., with darbepoetin alfa) offers added benefit requires dedicated comparative studies.

In addition, nanosized iron oxide nanoparticles possess dual effects of iron supplementation and anti-inflammation. They can downregulate ferritin, elevate transferrin saturation, and simultaneously inhibit the expression of the inflammatory cytokine TNF-α, thereby effectively correcting anemia in CKD mouse models while circumventing the risks of inflammatory activation and iron overload associated with conventional iron supplementation. However, this agent is still in the basic research stage and has not yet been applied clinically; its clinical efficacy and safety require further validation (Hsiao et al., 2023).

#### 5.4.2 CKD-MBD-targeted agents

A prospective study showed that treatment with the calcium-sensing receptor agonist cinacalcet for more than 6 months in CKD patients not only improved calcium-phosphorus parameters and PTH levels but also effectively reduced ERI and alleviated anemia (Weng et al., 2025). Vitamin D supplementation was also found to improve anemia parameters in rats by downregulating hepcidin expression (Xu et al., 2025). In addition, HIF-PHIs have been shown to improve anemia while reducing FGF23, suggesting that FGF23 is a potential therapeutic target for anemia that may be considered in the future (Wang et al., 2025), through indirectly ameliorating anemia as an adjunctive therapy by improving EPO resistance.

#### 5.4.3 Sodium-glucose cotransporter 2 inhibitors

SGLT-2i inhibit renal tubular reabsorption of glucose and promote urinary glucose excretion, and were therefore initially developed as glucose-lowering agents. Emerging evidence indicates that SGLT-2i confer beneficial effects on renal anemia, in addition to slowing CKD progression and reducing cardiovascular and sepsis-related events (Bhatt et al., 2021; Nuffield Department of Population Health Renal Studies Group, 2022; van der Meer et al., 2005; Koshino et al., 2023b). Post-hoc analyses of two large CKD-specific trials—the Canagliflozin and Renal Endpoints in Diabetes with Established Nephropathy Clinical Evaluation (CREDENCE) trial and the Dapagliflozin in Patients with Chronic Kidney Disease (DAPA-CKD) trial—have further reinforced this association, providing direct and quantifiable evidence for the erythropoietic effects of SGLT-2i (Koshino et al., 2025; Oshima et al., 2020; Koshino et al., 2023b).

The CREDENCE trial enrolled 4,401 patients with type 2 diabetes and CKD and an eGFR of 30–90 mL/min/1.73 m^2^, with a median follow-up of 2.6 years. Results showed that the canagliflozin group had a mean hemoglobin level 7.1 g/L higher than that of the placebo group (95% CI 6.4–7.8), and a 35% reduction in the composite endpoint of anemia events or initiation of therapy (HR 0.65, 95% CI 0.55–0.77). With respect to individual anemia-related endpoints, canagliflozin reduced the risk of anemia events, initiation of iron therapy, and initiation of ESA therapy by 42% (HR 0.58, 95% CI 0.47–0.72), 36% (HR 0.64, 95% CI 0.52–0.80), and 35% (HR 0.65, 95% CI 0.46–0.91), respectively (Oshima et al., 2020). Notably, the hemoglobin-raising effect of canagliflozin was independent of baseline iron status, with consistent efficacy observed in both iron-deficient and non-iron-deficient patients (Koshino et al., 2025; Oshima et al., 2020). The DAPA-CKD trial further extended these findings. This trial enrolled patients with CKD stages 2–4 and an eGFR of 25–75 mL/min/1.73 m^2^. Post-hoc analysis showed that among patients with baseline anemia, the anemia correction rate was 53.3% in the dapagliflozin group, significantly higher than 29.4% in the placebo group (HR 2.29, 95% CI 1.96–2.68); among those without baseline anemia, the incidence of new-onset anemia decreased from 23.7% to 10.4% (HR 0.39, 95% CI 0.31–0.48), and this effect was consistent across patients with and without type 2 diabetes (Koshino et al., 2023b).

Taken together, these results support a reproducible clinical effect of SGLT-2i on erythropoiesis. However, as the above analyses are all *post hoc* analyses of large RCTs, these agents should currently be regarded as foundational CKD therapy with additional anemia-related benefits, rather than as dedicated anti-anemia agents that replace ESAs, HIF-PHIs, or iron preparations. Moreover, the mechanisms underlying the erythropoiesis-enhancing effect of SGLT-2i have not been fully elucidated, and are hypothesised to involve promotion of EPO synthesis, as well as improvement in inflammation and iron metabolism (Yaribeygi et al., 2022). To date, these agents have not been specifically approved for the treatment of renal anemia, and their adverse effects, including urinary tract infection and diabetic ketoacidosis, as well as eGFR-related restrictions, should also be taken into consideration in clinical decision-making (Bhatt et al., 2021; Nuffield Department of Population Health Renal Studies Group, 2022).

#### 5.4.4 Traditional Chinese medicine

A meta-analysis showed that adding traditional Chinese medicine (TCM) with spleen-strengthening and kidney-tonifying effects to conventional therapy can effectively improve anemia parameters, lower hepcidin and ferritin levels, and enhance renal function in the treatment of renal anemia (Li et al., 2020a). Compound TCM formulas, single herbs, and their extracts can intervene in renal anemia through multiple targets: upregulating HIF-2α to promote EPO production, inhibiting the JAK2-STAT3 pathway to regulate iron metabolism (Wang et al., 2020; Li et al., 2023), and blocking the NF-κB and TGF-β/Smad pathways to delay renal fibrosis, thereby simultaneously improving EPO deficiency and EPO resistance (Huang et al., 2024).

The compound TCM preparation Shengxuening can increase SCF levels, activate the SCF/c-KIT/PI3K/AKT signaling pathway, promote the proliferation and differentiation of HSCs, and concurrently reduce inflammatory responses, thereby correcting renal anemia, with the advantages of high safety and few gastrointestinal side effects (Zeng et al., 2024; Zeng et al., 2020). In addition, single TCM herbs and their active components also exhibit anemia-improving effects. Extracts from *Gynura Procumbens* can alleviate inflammatory infiltration by inhibiting xanthine oxidase activity, reduce renal tubular cell apoptosis, ameliorate renal fibrosis, and promote HIF-2α and EPO expression (Li et al., 2024). Tangerine peel and its component nobiletin can activate EPOR and inhibit erythrocyte apoptosis by enhancing the JAK2/STAT5 pathway, thus treating renal anemia (Liu et al., 2025a).

However, there are notable limitations in the TCM field. Existing studies are predominantly single-center, small-sample RCTs, lacking international multicenter, large-sample evidence-based data. Moreover, the quality control and standardization systems for TCM are still absent, leaving a considerable gap before widespread international application. In addition, the complex composition of TCM formulas, the unclear identity of active monomers and their targets, and the insufficient depth of mechanistic research on TCM treatment of anemia remain major challenges.

## 6 Discussion

The pathophysiological mechanisms of renal anemia center on EPO deficiency and EPO resistance. Current exploration of this disease has extended to underlying molecular mechanisms and microenvironmental crosstalk. Figure 4 presents a schematic dual-axis model that visually depicts the interplay between these two pathogenic pathways and outlines the complete clinical management chain from screening and correction of reversible triggers, through pharmacological intervention, to follow-up monitoring, along with the corresponding therapeutic agents. Future progress in this field will require refined regulation of EPO synthesis and erythroid responsiveness, as well as a focus on stratified management based on patient heterogeneity.

**FIGURE 4:**
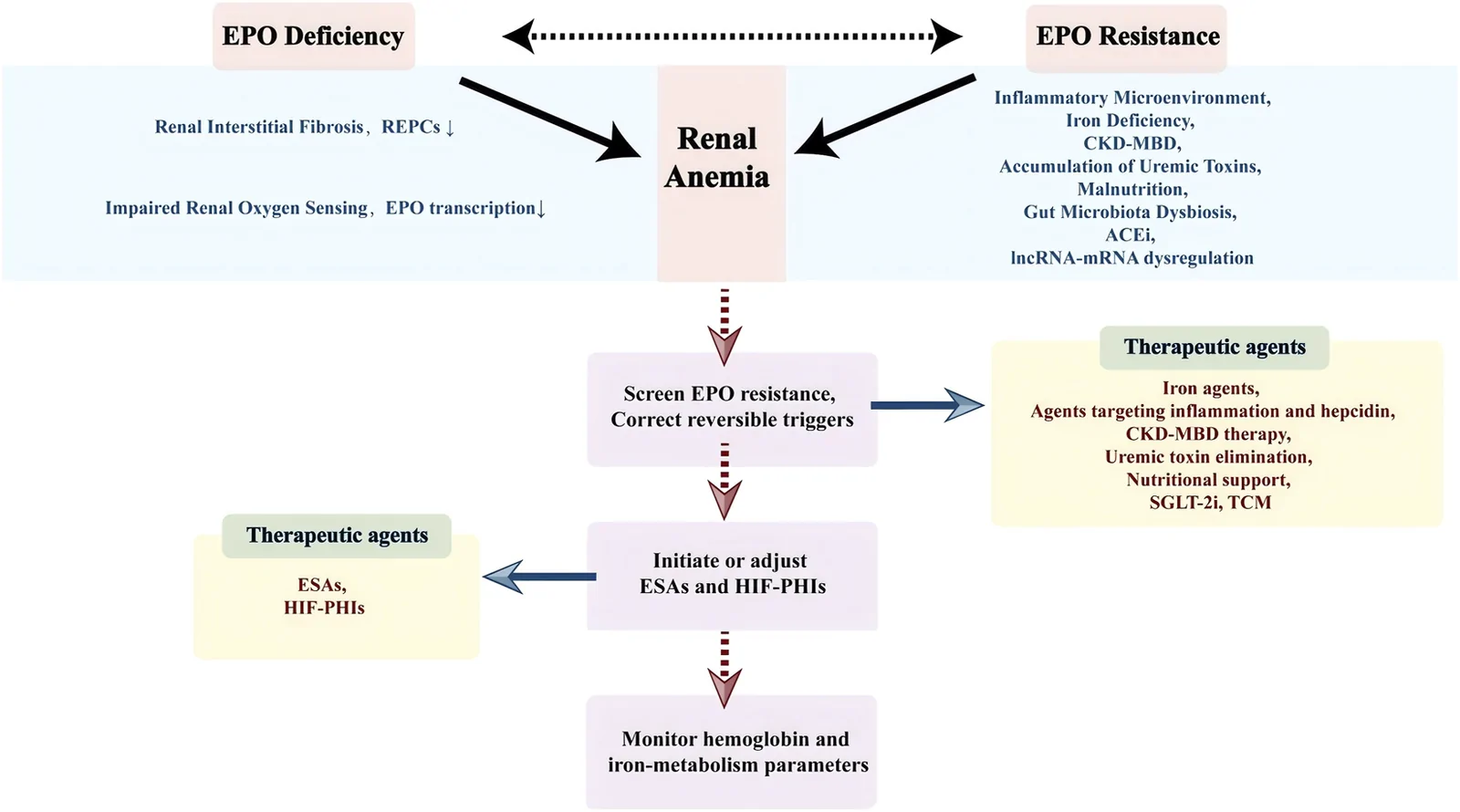
Recapitulative overview of renal anemia: dual pathogenic axes and step-wise clinical management. This concluding schematic summarizes the core conceptual framework of the present review. Renal anemia is jointly driven by EPO deficiency and EPO resistance, with bidirectional crosstalk between these two pathogenic axes. Renal interstitial fibrosis and impaired renal oxygen sensing contribute to EPO deficiency. Multiple uremia-related insults including inflammatory microenvironment, iron deficiency, CKD-MBD, uremic toxin accumulation, malnutrition, gut microbiota dysbiosis, ACEi exposure and lncRNA-mRNA dysregulation promote EPO resistance. Once renal anemia occurs, the step-wise clinical workflow includes screening for EPO resistance and correction of reversible triggers, initiation or dose adjustment of ESAs and HIF-PHIs, and regular monitoring of hemoglobin and iron status markers throughout anti-anemia therapy. Corresponding therapeutic agents for each clinical intervention are displayed. Solid black arrows denote pathogenic pathways; dotted black two-way arrow indicates bidirectional crosstalk between EPO deficiency and EPO resistance; red dotted arrows represent sequential clinical management workflow; solid blue arrows indicate corresponding therapeutic agents for clinical interventions. EPO, Erythropoietin; REPCs, renal erythropoietin-producing cells; CKD-MBD, chronic kidney disease-mineral and bone disorders; ACEi, angiotensin-converting enzyme inhibitors; lncRNAs, long non-coding RNAs; ESAs, erythropoiesis-stimulating agents; HIF-PHIs, hypoxia-inducible factor prolyl hydroxylase inhibitors; SGLT-2i, sodium-glucose cotransporter 2 Inhibitors; TCM, traditional Chinese medicine.

With respect to EPO synthesis, traditional concepts are facing renewal. Classical theory holds that hypoxia induces HIF-2α to drive *Epo* transcription. Accordingly, the renal medulla, which is under physiological hypoxic conditions, should theoretically possess the potential for EPO synthesis. However, recent studies have found that although pharmacological PHD inhibitors (HIF-PHIs) can enhance HIF-2α stability throughout the kidney, they fail to activate EPO expression in fibroblasts of the deep medulla. Yet, genetic knockout of PHD not only stabilizes HIF-2α but also successfully induces EPO expression in wide renal regions, including the deep medulla. This suggests the existence of unknown, cell-type-specific *Epo* transcriptional regulatory networks. Elucidation of the relevant mechanisms may provide entirely new directions for targeted therapy of renal anemia (Firmke et al., 2025).

With respect to ameliorating EPO resistance, treatment strategies are becoming increasingly diversified, but the evidence chain for long-term efficacy and safety remains incomplete. Drugs originally indicated for other disease areas, such as SGLT-2 inhibitors and cinacalcet, have also been shown to alleviate EPO resistance. Novel anemia agents, including hepcidin-targeted agents, IL-6 monoclonal antibodies and nano-sized iron oxide nanoparticles, are gradually emerging, but most related studies are still at the basic experimental or early exploratory stage, and their clinical efficacy and safety require validation through longer-term, larger-sample studies. In addition, the therapeutic value of traditional Chinese medicine in renal anemia continues to be explored, but future efforts should promote standardization of clinical research and deepen mechanistic investigations.

For clinicians, achieving precision medicine remains a core challenge in future clinical practice. It is necessary to establish a standardized screening process for reversible causes of EPO resistance, incorporating markers of inflammation, iron metabolism, CKD-MBD, and nutrition into routine evaluations. Reversible factors should be addressed as a priority, after which ESAs, HIF-PHIs, or adjunctive targeted therapies may be individually selected, with the aim of guiding personalized combinations and dose adjustments of currently available drugs.
